# Analysis of the Association between Protein Intake and Disability-Adjusted Life Year Rates for Alzheimer’s Disease in Japanese Aged over 60

**DOI:** 10.3390/nu16081221

**Published:** 2024-04-19

**Authors:** Kazuki Fujiwara, Takayuki Tanaka, Hisamine Kobayashi, Kenji Nagao, Kazuko Ishikawa-Takata

**Affiliations:** 1 Research Institute for Bioscience Products & Fine Chemicals, Ajinomoto Co., Inc., Kanagawa 210-8681, Japan; 2 Business Strategy & Planning Department, Ajinomoto Co., Inc., Kanagawa 210-8681, Japan; 3 Faculty of Applied Biosciences, Tokyo University of Agriculture, Tokyo 156-8502, Japan

**Keywords:** protein, Alzheimer’s disease, disability-adjusted life years, global burden of disease, national health and nutrition survey

## Abstract

With advancements in medical technology, the structure of disease is shifting from acute illnesses to chronic conditions, such as Alzheimer’s disease (AD). Consequently, there is an escalating need for evaluations that discourse on the potential effects on healthy life years, as well as disease onset. We aimed to evaluate the associations with AD disability-adjusted life year (AD-DALY) rates and protein intake by sex and age group. For the analysis, we used representative values for males and females in their 60s and aged over 70, extracted from the public dataset of the Global Burden of Disease Study and the National Health and Nutrition Survey in Japan, covering the years 1990 to 2019. In order to evaluate the association between AD-DALY rates and protein intake, we analyzed correlations and stratified multiple regression models. Additionally, we simulated alterations in AD-DALY rates associated with changes in protein intake by utilizing stratified multiple regression models. AD-DALY rates and protein intake indicated significant negative correlations across all sex and age groups. In stratified multiple regression models, significant associations were found between higher protein intake and lower AD-DALY rates in females. In the simulation, when protein intake was increased to 1.5 g/kg/day, AD-DALY rates decreased by 5–9 percent compared with 2019. However, the association between intake of animal and plant protein and AD-DALY rates were found to vary based on sex and age group. The present study suggests the possibility to improve AD-DALY rates by increasing population average protein intake levels in a recommended range.

## 1. Introduction

Dementia is forecasted to reach 110 million people globally by 2050, with the associated societal and economic losses potentially exceeding USD 604 billion [1]. According to the 2019 National Livelihood Survey in Japan, dementia is cited as the principal cause of individuals needing care, accounting for 24.3 percent of cases [2], marking it as a societal issue. About 60 percent of all dementia cases are reported as Alzheimer’s disease (AD), with the reported incidence rate being 13 percent for those aged over 65 and 45 percent for those aged over 85 [3,4].

According to the review examining the relations between diet and cognitive decline, the effects of the intake of several nutrients and of dietary patterns were reported [5]. Intake of polyphenols, B vitamins, and long-chain fatty acids was shown to be effective in preventing cognitive decline. In addition, various dietary patterns, such as the Mediterranean diet and the Dietary Approaches to Stop Hypertension (DASH) diet, might help to prevent cognitive decline. The World Health Organization (WHO) recommends maintaining a balanced and nutritious diet to mitigate the risk of cognitive decline and dementia [6].

Excessive intake of energy increases the risk of AD [7]. However, the impact of the intake of each macro-nutrient on the risk of AD is still unclear. In terms of protein, it was indicated that increased protein intake decreases risks of cognitive decline and AD [8,9,10], although the outcome measures and methods used to assess them vary among research studies. Contrarily, a cross-sectional study using the National Health and Nutrition Examination Survey in the United States did not find an association between protein intake and memory disorders [11]. However, the effect of macro-nutrient balance may be different depending on the usual intake in a specific population; therefore, a study using data of the Japanese would be needed.

As a result of the development of medical technology, the structure of disease is shifting from acute illnesses to chronic conditions, such as AD. Accordingly, there is an escalating need for evaluations that discourse on the potential effects on healthy life years, as well as disease onset. Lopez proposed disability-adjusted life year (DALY) rates et al. [12] and quantify the loss of time due to death or disability to evaluate the healthy life years of a population. An increase of 1 DALY is denoted as one year of life lost due to disease or disability. In addition to being adopted by insurance outcome metrics by the WHO and World Bank [13], DALY rates are used to study the association between nutritional status and healthy life years in different countries. DALY rates have been calculated for each distinct combination of disease, country, survey year, sex, and age groups, so they have been applied to research from various perspectives. Specifically, research was reported on systematic analyses of protein–energy malnutrition [14] and vitamin A deficiency [15] globally and on the association between salt or vegetable intake and DALYs in Japan [16,17]. Regarding protein intake, although research studies have shown a decreased risk of AD, its association with AD-DALY rates has not been evaluated [8,9,10]. In this study, we aimed to evaluate the association between protein intake and AD-DALY rates in Japan.

## 2. Materials and Methods

### 2.1. Data Source

We used two datasets which are generally available to the public and do not include any personally identifying information.

#### 2.1.1. Global Burden of Diseases, Injuries, and Risk Factors Study Dataset

From the Global Burden of Diseases, Injuries, and Risk Factors Study (GBD) dataset on the official GBD website [13], AD-DALY rates and the Socio-Demographic Index (SDI) were obtained for Japanese males and females in their 60s and over 70 years old from 1990 to 2019. AD-DALY rates in this paper are the sum of years lived with disability and years of life lost related to AD per 100,000 people, calculated annually for each sex and age group in Japan. The SDI is an annually calculated geometric mean ranging from 0 to 1 for each country which considers factors such as the total fertility rate of those under 25 years old, the average education level for individuals aged over 15, and lag distributed income per capita. This index was used as a confounding variable to account for intergenerational effects.

#### 2.1.2. National Health and Nutrition Survey Dataset for Japan

We used the average values for four demographic groups, i.e., males and females in their 60s and over 70 years old, obtained from the public database of the National Health and Nutrition Survey (NHNS) in Japan [18] covering 1990 to 2019. The public database includes only population average data for each sex and age group. Then, population average data on body weight and intake of energy, protein, animal protein, and plant protein were used. The number of participants in the NHNS in Japan in each year according to the age group was as follows: 544 to 3088 for females in their 60s, 502 to 2763 for male in their 60s, 640 to 3897 for female over 70 years old, and 393 to 3015 for male over 70 years old. To eliminate any effect of changes in body weight between 1990 to 2019, energy and the intake of each protein were calculated as the average intake divided by the average body weight. For example, the average protein intake among males in their 60s in 2019 was 75.2 g, and the average body weight was 67.3 kg; then, the average protein intake per body weight was calculated as 75.2/67.3 = 1.117 g/kg.

### 2.2. Statistical Analysis

In this study, associations between AD-DALYs and protein intake were explored by univariate analysis and evaluated by using multiple regression models and simulations. 

#### 2.2.1. Univariate Analysis

Data are shown as means and standard deviations (SDs) for the period 1990 to 2019 for each sex and age group. Pearson correlation coefficients between AD-DALYs and other variables for each sex and age group were calculated.

#### 2.2.2. Evaluation Using Multiple Regression Models

For each sex and age group, multiple regression models were developed, using AD-DALY rates as the dependent variable and energy and SDI as the confounding variables. In Model I, protein intake was contained as an independent variable. Estimates of AD-DALY rates in Model I for any given combination of year y, sex, and age are denoted by $E_{y}^{I}\left(sex,\;age\right)={X_{y}^{I}\left(sex,\;age\right)}^{T}W_{I}\left(sex,\;age\right)$. Independent variables ($X_{y}^{I}\left(sex,\;age\right)$) and partial regression coefficient ($W_{I}\left(sex,\;age\right)$) vectors were set as intercept, protein, energy, and SDI terms, respectively.

$$X_{y}^{I}\left(sex,\;age\right)=\left[\begin{array}{c} \begin{array}{c} 1\\ x_{y}^{p}(sex,\;age) \end{array}\\ x_{y}^{e}(sex,\;age)\\ x_{y}^{s}(sex,\;age) \end{array}\right],W_{I}\left(sex,\;age\right)=\left[\begin{array}{c} \begin{array}{c} w_{I}^{i}(sex,\;age)\\ w_{I}^{p}(sex,\;age) \end{array}\\ w_{I}^{e}(sex,\;age)\\ w_{I}^{s}(sex,\;age) \end{array}\right]$$

Model II incorporated both animal protein intake and plant protein intake as independent variables. Model II estimate is defined by $E_{y}^{II}\left(sex,\;age\right)={X_{y}^{II}\left(sex,\;age\right)}^{T}W_{II}\left(sex,\;age\right)$. Independent variables ($X_{y}^{II}\left(sex,\;age\right)$) and partial regression coefficient ($W_{II}\left(sex,\;age\right)$) vectors were set as intercept, protein, energy, and SDI terms, respectively.

$$X_{y}^{II}\left(sex,\;age\right)=\left[\begin{array}{c} \begin{array}{c} \begin{array}{c} 1\\ x_{y}^{ap}(sex,\;age) \end{array}\\ x_{y}^{pp}(sex,\;age) \end{array}\\ x_{y}^{e}(sex,\;age)\\ x_{y}^{s}(sex,\;age) \end{array}\right],W_{II}\left(sex,\;age\right)=\left[\begin{array}{c} \begin{array}{c} w_{II}^{i}(sex,\;age)\\ \begin{array}{c} w_{II}^{ap}(sex,\;age)\\ w_{II}^{pp}(sex,\;age) \end{array} \end{array}\\ w_{II}^{e}(sex,\;age)\\ w_{II}^{s}(sex,\;age) \end{array}\right]$$

The intake of each protein type input into models was in units of 0.1 g/kg/day (daily intake per body weight).

#### 2.2.3. Model-Based Simulation

By using developed multiple regression models, the effects of fluctuating protein intake on AD-DALY rates were simulated by sensitivity analysis [19]. Expanding the formula for Model I estimates ($E_{y}^{I}\left(sex,\;age\right)$), the only term involving protein is $w_{I}^{p}(sex,\;age)\;x_{y}^{p}(sex,\;age)$. Therefore, when protein intake is increased by $\Delta x_{y}^{p}(sex,\;age)$, the simulated values for AD-DALY rates are ${E_{y}^{I}\left(sex,\;age\right)}^{\prime }=E_{y}^{I}\left(sex,\;age\right)+w_{I}^{p}\left(sex,\;age\right)\Delta x_{y}^{p}(sex,\;age).$ Similarly, in Model II, when increasing the intake of $\Delta x_{y}^{ap}(sex,\;age)$ for animal protein and $\Delta x_{y}^{pp}(sex,\;age)$ for plant protein, the simulated values for AD-DALY rates are ${E_{y}^{II}\left(sex,\;age\right)}^{\prime }=E_{y}^{II}\left(sex,\;age\right)+w_{II}^{ap}\left(sex,\;age\right)\Delta x_{y}^{ap}\left(sex,\;age\right)+w_{II}^{pp}\left(sex,\;age\right)\Delta x_{y}^{pp}(sex,\;age).$ In these simulations, we investigated the impact of AD-DALY rates when increased protein intake, including that of plant and animal protein, is the only focus. In all simulations, confounding variables such as energy and SDI were set as constant values at the 2019 level. 

To prevent sarcopenia in the elderly, it is suggested that they consume 1.0–1.5 g/kg/day of protein [20]. In Model I, protein intake was simulated to increase within this range for the most recent 2019 dataset. In other words, the constraint equation for protein intake is 1.0 $\leq x_{2019}^{p}\left(sex,\;age\right)+\Delta x_{2019}^{p}\left(sex,\;age\right)\leq$ 1.5 [g/kg/day]. Model II explored situations in which animal and plant protein increased by 0.05 g/kg/day from the actual intake in 2019. We simulated to change the intake of both animal and plant protein while ensuring the total protein intake within the suggested upper level of 1.5 g/kg/day. Then, the constraint equation for animal and plant protein intake is 1.0 $\leq x_{2019}^{ap}\left(sex,\;age\right)+\Delta x_{2019}^{ap}\left(sex,\;age\right)+x_{2019}^{pp}\left(sex,\;age\right)+\Delta x_{2019}^{pp}\left(sex,\;age\right)\leq$ 1.5 [g/kg/day].

#### 2.2.4. Analysis Environment and Significance Threshold

All analysis were conducted by Python, version 3.7.3. The significance threshold was set to *p*-value < 0.05, and the trend was set to *p*-value < 0.1.

## 3. Results

### 3.1. Univariate Analysis

Table 1 shows mean, standard deviation, and correlation coefficient for each variable by sex and age group from 1990 to 2019. The mean AD-DALY rates for males and females in their 60s were 495.303 and 510.120, respectively. Those aged over 70 showed higher AD-DALY rates than their counterparts in their 60s for both males and females (males: 4295.484; females: 6625.386). The AD-DALY rates indicated significant positive correlations with the SDI across all sex and age groups. Significant negative associations were observed between AD-DALY rates and energy intake, with correlation coefficients ranging from −0.568 to −0.871 (*p* < 0.001 for each). Protein, animal protein, and plant protein intake showed significant negative associations, with correlation coefficients between −0.473 and −0.891 for all sex and age groups (*p* < 0.001 for each). Correlation coefficients between AD-DALY rates and energy or protein intake were stronger among individuals in their 60s than those aged over 70 for both sexes.

### 3.2. Evaluation using Multiple Regression Models

The coefficients of determination ($R^{2}$) in Model I ranged from 0.785 to 0.972 (Table 2). The partial regression coefficients (β) for protein were negative across all sex and age groups, with the lowest value being found for females aged over 70 (β = −418.062, *p* = 0.002). Based on the partial regression coefficients for females aged over 70, increased protein intake by 0.1 g/kg/day was associated with decreased AD-DALY rates by 418.062, indicating lower risk of worsening health status. The effect of protein intake was significant in females (in their 60s: *p* = 0.017; aged over 70: *p* = 0.002), with only a trend being observable in males (in their 60s: *p* = 0.078; aged over 70: *p* = 0.057). 

The $R^{2}$ in Model II ranged from 0.785 to 0.972. Animal protein intake had a significant negative association with AD-DALY rate in females aged over 70 (β = −487.261, *p* = 0.044) and a negative trend in males in their 60s (β = −18.861, *p* = 0.069). A significant negative association was observed only in males aged over 70 (β = −605.898, *p* = 0.038) regarding plant protein intake.

### 3.3. Model-Based Simulation

**Figure 1:**
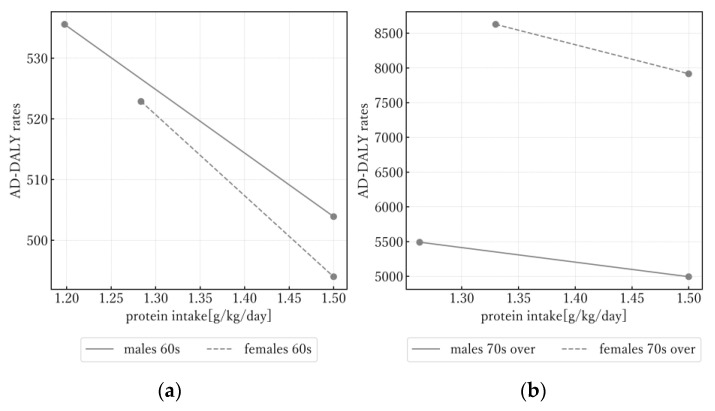
Simulation of Alzheimer’s disease disability-adjusted life year rates with increase in 2019 protein intake. Results for individuals in their 60s are shown in (**a**) and for individuals aged over 70 in (**b**). AD-DALY: Alzheimer’s disease disability-adjusted life year.

Figure 1 shows the estimated AD-DALY rates when protein intake was varied by using Model I. A simulation was conducted by increasing protein intake from the actual protein intake values in 2019 (1.33 for males and 1.34 for females in their 60s; 1.28 for males and 1.31 for females aged over 70) to the suggested upper limit of 1.5 g/kg/day. AD-DALY rates became about 5–9 percent lower than the rates in 2019 for all sex and age groups.

**Figure 2:**
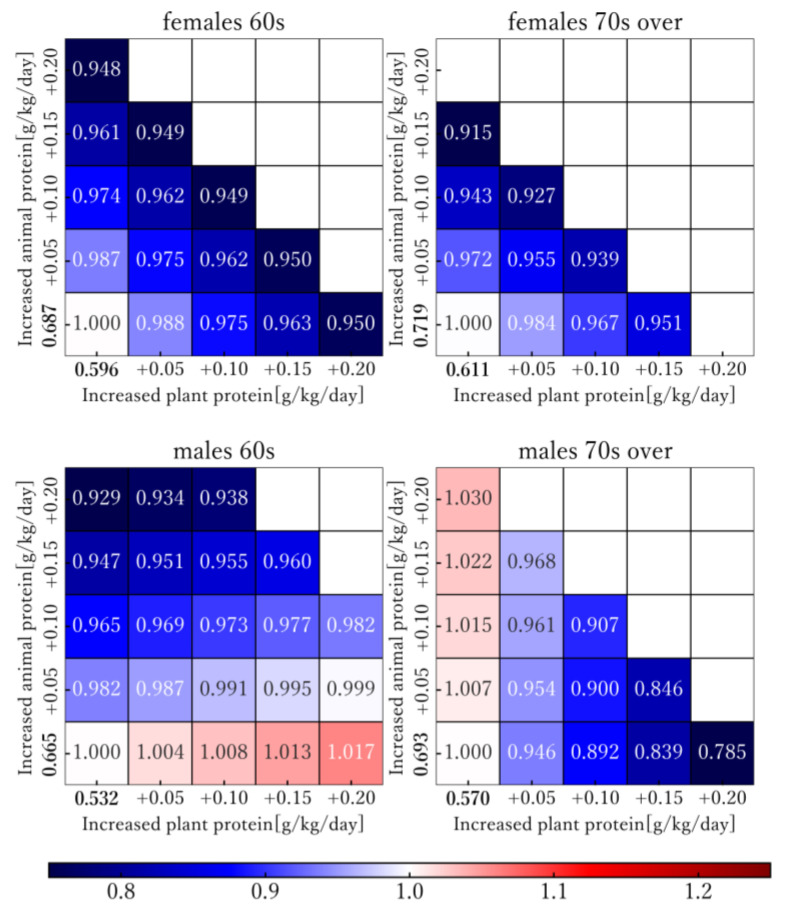
Simulation of Alzheimer’s disease disability-adjusted life year rates with increase in 2019 animal and plant protein intake. The actual measured animal and plant protein intake values for 2019 by sex and age group are shown in bold. The values in the heatmap are shown in different colors: blue for relatively low values, white for values at the same level, and red for high values. Heatmaps show the estimated Alzheimer’s disease disability-adjusted life year (AD-DALY) rates for 25 patterns, based on a 0.05 g/kg/day increase in intake of animal and plant protein, by sex and age groups. White boxes represent the current AD-DALY rates as the reference (1.000) for the current levels of animal and plant protein consumed.

Figure 2 shows the simulation results of AD-DALY rates using Model II when animal and plant protein intake was varied. We explored situations in which animal and plant protein increased by 0.05 g/kg/day from the actual intake in 2019. When the total of animal and plant protein intake exceeded 1.5 g/kg/day, boxes are shown as blank. The values in the heatmap denote relative ratios (${E_{y}^{II}\left(sex,\;age\right)}^{\prime }/E_{y}^{II}\left(sex,\;age\right)$) when AD-DALY rates in 2019 are set to 1.

For males in their 60s, the AD-DALY rates were high with additional plant protein and low with increased animal protein intake. In contrast, the AD-DALY rates for males aged over 70 were high with adding animal protein and low with additional plant protein intake. The maximum decrease in AD-DALY rates, 21.5 percent, was found when only plant protein was increased by 0.20 g/kg/day and animal protein intake was not varied in males aged over 70. As one AD-DALY decrease in the rates is equivalent to one year of healthy life regained per 100,000 people, this rate of reduction corresponds to approximately 1210 years per 100,000 people. In females, the estimated AD-DALY rates decreased as animal or plant protein intake increased. In females, the greatest decreases were found when animal protein was increased (0.20 and 0.15 g/kg/day for females in their 60s and aged over 70, respectively) and plant protein intake was not varied.

## 4. Discussion

In the present study, we conducted an analysis based on 30 years of public datasets and found that an increase in population average protein intake was associated with lowered AD-DALY rates. The simulation results indicate that increasing protein intake up to 1.5 g/kg/day, which is the upper limit of the recommended amount for preventing sarcopenia in the elderly [20], presents the possibility to lower AD-DALY rates by 5–9 percent compared with the actual rates in 2019. The results relative to the combination of animal and plant protein varied across sex and age groups.

The present results show that high levels of population average protein intake were associated with lower AD-DALY rates. These results support prior research suggesting that a higher protein–energy ratio was associated with reduced risks of onset of mild cognitive impairment [8] and subjective cognitive function [9] compared with the reference group of the lowest protein–energy ratio. Additionally, in the elderly, it has been reported that individuals with a high protein intake show a lower accumulation of amyloid β, a contributor to AD, in the brain [21]. This finding could be one of the potential reasons for the present negative correlation between protein intake and AD-DALY rates. However, it remains uncertain whether protein intake directly affects AD-DALY rates. High protein intake was associated with an increase in lean body mass and muscle strength [22]. An observation study of 3.6 years suggested that elderly individuals with greater muscle strength had a lower risk of AD [23]. Accordingly, there is a possibility that protein intake affects AD-DALY rates through its influence on muscle strength. Also, it has been reported that protein and amino acid intake may be related to cognitive function through increased levels of physical activity and enhanced sleep quality [24].

Although correlations between protein intake and AD-DALY rates have been suggested, determining the recommended amount to decrease health loss attributable to AD remains a complex task. In a review by Glenn et al. [24], protein and amino acid intake was associated with cognitive function, but further research is needed to propose these recommended amounts. In addition, the present study only analyzed linear relationships between protein intake and AD-DALY rates, but this linear relationship may not continue to be valid when protein intake increases too much. Therefore, the present simulations were conducted within the recommended range of protein intake (1.0–1.5 g/kg/day) for the elderly to prevent sarcopenia [20]. In a study evaluating the risk of cognitive decline in groups stratified by protein–energy ratio, nonlinear associations were confirmed [25]. The study found that a moderate energy ratio of 16.8–21.6 percent was associated with the lowest risk, while protein–energy ratios below 11.0 percent or over 21.6 percent were associated with increased risk. Roberts et al. [8] also found that the risk of mild cognitive dysfunction was lower in groups with a protein–energy ratio of 16–18 percent and 19–20 percent compared with the group with a protein–energy ratio lower than 16 percent. In the present datasets, an protein–energy ratio of 21.6 percent is equal to 1.7–1.8 g/kg/day of protein intake. Consequently, within the range used in this simulation, it might be possible to assume a linear relationship and suggest that higher population average protein intake is associated with lower AD-DALY rates.

Recommended protein intake is often determined in terms of both amount per body weight and protein–energy ratio. We used the amount of protein intake per body weight according to the recommended range of protein intake for the elderly to prevent sarcopenia [20]. In the Dietary Reference Intake in Japan, the provisional dietary goal for preventing lifestyle-related diseases linked to protein intake is shown as 15 to 20 percent protein–energy ratio [26]. This level of protein intake is consistent with the protein–energy ratios that have the lowest risk of cognitive decline [8,25]. The Japanese often have lower body weight and higher energy requirements due to a lower prevalence of obesity [27] and higher physical activity levels [28]. Then, protein levels per body weight often exceed the value calculated from energy intake at the recommended protein–energy ratio. In the present datasets, a protein–energy ratio of 20 percent is almost 1.7 g/kg/day, which exceeds the upper limit we used in the present study. However, we could not examine the upper limit of protein intake to improve AD-DALY rates because we only used population average data. 

The simulations found a trend towards lower AD-DALY rates with additional intake of either animal or plant protein in females in their 60s and over 70 years old. Males showed a trend towards lower AD-DALY rates only when they consumed additional animal protein in their 60s and plant protein when aged over 70. A four-year follow-up study suggested that when substituting 5 percent of energy from animal protein with plant protein, the risk of declining subjective cognitive function decreases by about 16 percent [9]. However, because the above study was conducted in the USA, the actual proportion of animal protein was greater than that in the present study. The effect of the increase in animal or plant protein or the substitution of the protein source is thought to differ depending on the baseline protein intake. In addition, depending on the kind of animal protein, the effect of increasing animal protein might be different. Previous studies have suggested that the effects of whole milk and low-fat milk or processed and unprocessed meat on cognitive function are different [29,30]. From these perspectives, further research is needed to elucidate associations between animal and plant protein intake and AD-DALY rates.

The present results show different relations between protein intake and AD-DALY rate among sex or age groups. One reason for this difference might be caused by the prevalence of AD by sex and age. According to the Patient Survey in 2020 in Japan [31], the numbers of male patients with AD were 8 thousand and 211 thousand for individuals in their 60s and aged over 70, respectively. In females, these were 11 thousand and 562 thousand for individuals in their 60s and aged over 70, respectively. This means that females over 70 years old are more likely to have AD. In addition, the average intake of total protein and plant protein per body weight per day is greater in females than in males. The simulation of increased animal and plant protein with AD-DALY rates might differ because of the actual intake. There may be a specific, appropriate balance of animal and plant protein; however, we were not able to determine it in the present study.

This study has the following three limitations: First, the study uses population averages, which are collected annually, because public data contain only population averages, not individual data. Therefore, these data may not correctly evaluate individual differences. Future research should use individual data and perform analyses adjusted for individual differences and other confounding factors. Second, there could be potential effects on AD-DALY rates from independent risk factors that were not included in the models. Confounding variables, such as exercise [32], drinking habits [33], and fat intake [34], which are considered risk factors for AD, and DALY-related to comorbidities would have to be included. Higher protein intake may increase the intake of other nutrients associated with AD; however, the present analysis did not examine the effects of the intake of other nutrients. In addition, the present study standardized protein intake per body weight. The differences in lean mass as a percentage of total body weight may affect the actual protein requirements [35]. However, the NHNS in Japan did not measure lean mass; therefore, we could not standardize protein intake by lean mass. The proportions of obesity (body mass index ≥ 25 kg/m^2^) were 28.3 ± 3.5%, 28.9 ± 4.1%, 26.6 ± 4.5%, and 22.9 ± 4.5% for females in their 60s, males in their 60s, females aged over 70, and males aged over 70, respectively. These proportions did not differ much by survey year, so we thought that the effect of obese participants on the population averages did not differ much by survey year. Finally, this study analyzed the association between population average protein intake and AD-DALY rates by using an annual dataset, but it was not able to assess causality. Moreover, although the SDI was used as a confounding variable, generational effects could not be eliminated.

## 5. Conclusions

In the present study, we analyzed the association between AD-DALY rates and protein intake by using population average data from public datasets and obtained the following findings:

- Significant associations were found between higher protein intake and lower AD-DALY rates after excluding the effect of energy intake and socio-demographic factors in females.
- In the simulation, when protein intake was increased to 1.5 g/kg/day, AD-DALY rates decreased by 5–9 percent compared with 2019. However, the association between intake of animal and plant protein and AD-DALY rates were found to vary based on sex and age group.

## Figures and Tables

**Table 1:** Summarized statistics and correlation coefficients for each sex and age group.

Variable	Age	Sex	Mean ± SD	Correlation Coefficient (*p*-Value)									
				SDI		Energy (kcal/kg/Day)		Protein (g/kg/Day)		Animal Protein (g/kg/Day)		Plant Protein (g/kg/Day)	
AD-DALY rates (years/100,000 people)	60s	Female	510.120 ± 18.160	0.820	(<0.001)	−0.825	(<0.001)	−0.877	(<0.001)	−0.801	(<0.001)	−0.875	(<0.001)
		Male	495.303 ± 29.845	0.913	(<0.001)	−0.871	(<0.001)	−0.863	(<0.001)	−0.768	(<0.001)	−0.891	(<0.001)
	70+	Female	6625.386 ± 1387.956	0.979	(<0.001)	−0.568	(0.001)	−0.718	(<0.001)	−0.473	(0.008)	−0.847	(<0.001)
		Male	4295.484 ± 772.953	0.954	(<0.001)	−0.756	(<0.001)	−0.784	(<0.001)	−0.632	(<0.001)	−0.864	(<0.001)
SDI	60s	Female	0.835 ± 0.022	-		−0.850	(<0.001)	−0.896	(<0.001)	−0.727	(<0.001)	−0.949	(<0.001)
		Male	0.835 ± 0.022	-		−0.907	(<0.001)	−0.854	(<0.001)	−0.704	(<0.001)	−0.925	(<0.001)
	70+	Female	0.835 ± 0.022	-		−0.590	(<0.001)	−0.693	(<0.001)	−0.423	(0.002)	−0.845	(<0.001)
		Male	0.835 ± 0.022	-		−0.819	(<0.001)	−0.790	(<0.001)	−0.643	(<0.001)	−0.865	(<0.001)
energy (kcal/kg/day)	60s	Female	33.642 ± 1.532	-		-		0.876	(<0.001)	0.759	(<0.001)	0.899	(<0.001)
		Male	34.850 ± 1.962	-		-		0.953	(<0.001)	0.869	(<0.001)	0.965	(<0.001)
	70+	Female	33.318 ± 1.714	-		-		0.884	(<0.001)	0.798	(<0.001)	0.863	(<0.001)
		Male	33.532 ± 1.231	-		-		0.886	(<0.001)	0.816	(<0.001)	0.887	(<0.001)
protein (g/kg/day)	60s	Female	1.337 ± 0.081	-		-		-		0.943	(<0.001)	0.979	(<0.001)
		Male	1.329 ± 0.126	-		-		-		0.960	(<0.001)	0.975	(<0.001)
	70+	Female	1.311 ± 0.091	-		-		-		0.931	(<0.001)	0.952	(<0.001)
		Male	1.282 ± 0.089	-		-		-		0.959	(<0.001)	0.969	(<0.001)
animal protein (g/kg/day)	60s	Female	0.688 ± 0.032	-		-		-		-		0.854	(<0.001)
		Male	0.700 ± 0.058	-		-		-		-		0.874	(<0.001)
	70+	Female	0.667 ± 0.044	-		-		-		-		0.775	(<0.001)
		Male	0.662 ± 0.043	-		-		-		-		0.859	(<0.001)
plant protein (g/kg/day)	60s	Female	0.650 ± 0.052	-		-		-		-		-	
		Male	0.629 ± 0.072	-		-		-		-		-	
	70+	Female	0.645 ± 0.052	-		-		-		-		-	
		Male	0.621 ± 0.049	-		-		-		-		-	

AD-DALY rates: Alzheimer’s disease disability-adjusted life year rates. SDI: Socio-Demographic Index. SD: standard deviation. Means and SDs were calculated from 30 population average data from 1990 to 2019 (30 years, one population average datum per year) for each sex and age group. Correlation coefficients indicate Pearson correlation between AD-DALY rates and other factors of data from 1990 to 2019.

**Table 2:** Multiple regression models for Alzheimer’s disease disability-adjusted life years developed for sex and age groups.

Model	Age	Sex	$R^{2}$	Partial Regression Coefficient (*p*-Value)									
				Protein (g/kg/Day)		Animal		Plant		Energy (kcal/kg/Day)		SDI	
						Protein (g/kg/Day)		Protein (g/kg/Day)					
Model I	60s	Female	0.785	−13.352	(0.017)					−2.545	(0.288)	84.462	(0.637)
		Male	0.862	−10.472	(0.078)					3.045	(0.506)	973.778	(<0.001)
	70+	Female	0.972	−418.062	(0.002)					174.117	(0.005)	57802.130	(<0.001)
		Male	0.923	−209.643	(0.057)					156.256	(0.065)	33935.859	(<0.001)
Model II	60s	Female	0.785			−13.579	(0.262)	−12.990	(0.470)	−2.562	(0.319)	89.210	(0.757)
		Male	0.868			−18.861	(0.069)	4.500	(0.775)	2.346	(0.611)	1214.526	(0.001)
	70+	Female	0.972			−487.261	(0.044)	−283.141	(0.483)	165.932	(0.013)	59557.907	(<0.001)
		Male	0.929			83.305	(0.703)	−605.898	(0.038)	152.262	(0.066)	29770.322	(<0.001)

AD-DALY rates: Alzheimer’s disease disability-adjusted life year rates. SDI: Socio-Demographic Index. Multiple regression models were developed from the 30 population average data from 1990 to 2019 (30 years, one population average datum per year) for each sex and age group.

## Data Availability

Data are available in the public, open-access repository through web sites.
